# Metabolic Syndrome **and Metabolic Dysfunction-associated Steatotic Liver Disease **in Fibromyalgia

**DOI:** 10.5152/ArchRheumatol.2025.25004

**Published:** 2025-09-03

**Authors:** Chia-Chang Chen, Yi-Hsing Chen, Der-Yuan Chen, Sheng-Shun Yang, Kuo-Tung Tang

**Affiliations:** 1 Division of Gastroenterology and Hepatology, Department of Internal Medicine, Taichung Veterans General Hospital, Taichung, Taiwan; 2 Department of Post-Baccalaureate Medicine, College of Medicine, National Chung Hsing University, Taichung, Taiwan; 3 Program in Translational Medicine, National Chung Hsing University, Taichung, Taiwan; 4 Division of Allergy, Immunology and Rheumatology, Taichung Veterans General Hospital, Taichung, Taiwan; 5 Faculty of Medicine, National Yang Ming Chiao Tung University, Taipei, Taiwan; 6 Rheumatology and Immunology Center, China Medical University Hospital, Taichung, Taiwan; 7 College of Medicine, China Medical University, Taichung, Taiwan; 8 Kuang Tien General Hospital, Taichung, Taiwan

**Keywords:** Cardiovascular diseases, carotid intima-media thickness, fibromyalgia, metabolic dysfunction–associated steatotic liver disease, metabolic syndrome

## Abstract

**Background/Aims:**

Fibromyalgia (FM) is a chronic disease characterized by widespread pain. An increased cardiovascular risk has been suggested in these patients. The aim was to investigate the cardiovascular risk factors in these patients.

**Materials and Methods:**

Patients with primary FM consecutively were prospectively recruited from a tertiary medical center in Taiwan. As the control group, individuals without FM who had undergone a health checkup examination were recruited. Their traditional cardiovascular risk factors, carotid intima-media thickness (IMT), and the presence of metabolic syndrome were then determined. Metabolic dysfunction–associated steatotic liver disease (MASLD) and the severity of hepatic steatosis (the Saverymuttu and Hamaguchi scores) were both determined by sonography. Multivariate logistic and linear regression were used to compare groups of subjects.

**Results:**

A total of 66 FM patients and 116 controls were recruited. Between FM patients and controls, comparable cardiovascular risk factors were found, including carotid IMT and metabolic syndrome. FM patients had a higher proportion of central adiposity when compared with the controls, with an odds ratio of 6.1 (95% CI: 2.9, 13.1). Fibromyalgia patients had a more severe hepatic steatosis, if present, as determined by the Hamaguchi score. In female subjects, FM patients had a higher proportion of MASLD when compared with the controls, with an odds ratio of 2.8 (95% CI: 1.3, 6.1). The disease severity was associated with left IMT value and lipid blood levels in FM patients.

**Conclusion:**

Fibromyalgia patients had a higher proportion of central adiposity and more severe hepatic steatosis when compared with the controls. Those patients with more severe FM symptoms likely had a higher cardiovascular risk. Metabolic dysfunction–associated steatotic liver disease merits more attention in FM patients.

**Main Points**

- The cardiovascular risk in fibromyalgia patients remains elusive.
- Fibromyalgia patients had a higher proportion of central adiposity and more severe hepatic steatosis when compared with the controls.
- Fibromyalgia patients with metabolic syndrome might have more severe symptoms when compared with those patients without.

## Introduction

Fibromyalgia (FM) is common, with a 2% prevalence rate in the general population. It is characterized by widespread chronic pain.^1^ Other bothersome symptoms are sleep disorders, fatigue, and cognitive dysfunction. Due to the sedentary lifestyle^2^ and probable inflammatory activity in FM patients,^3^ an elevated cardiovascular risk is speculated. An increased cardiovascular risk in FM patients was suggested in some studies,^4^^-^^7^ while another study found no such association.^8^ In recent decades, metabolic syndrome is known to be associated with a higher risk of developing cardiovascular events.^9^ In addition, metabolic syndrome is often associated with the development of metabolic dysfunction–associated steatotic liver disease (MASLD), which may culminate in liver cirrhosis.^10^

Here, the aim was to comprehensively study the cardiovascular risk factors and burden in FM patients, specifically their metabolic syndrome, MASLD, and carotid intima-media thickness (IMT). The hypothesis is that (a) these risk factors are more common in FM patients than in the general population, and (b) the presence of such factors is associated with the severity of FM symptoms.

## Methods

### Study Participants

We prospectively recruited 66 outpatients with primary FM consecutively at the Division of Allergy, Immunology, and Rheumatology, Taichung Veterans General Hospital. Fibromyalgia was diagnosed according to either the 1990 (n = 56) or 2016 revised (n = 10) criteria of the American College of Rheumatology (ACR).^11^^,^^12^ For control subjects, 117 individuals who consecutively underwent a health checkup examination at the hospital from November 2020 to January 2021 were prospectively recruited. One subject diagnosed with FM was excluded from the controls. Written informed consent of these study subjects was obtained before the study in line with the Declaration of Helsinki. This study was approved by the Institutional Review Board of Taichung Veterans General Hospital (Approval No.: CE15319A, date: January 4, 2016; Approval No.: CE19281B, date: September 2, 2019; Approval No.: CE20279B, date: September 29, 2020).

### Clinical Assessments

To evaluate the disease severity of FM, the validated Chinese version of the revised Fibromyalgia Impact Questionnaire (FIQR) (Cronbach’s α: 0.95 in 103 Taiwanese FM patients; Whei-Mei Shih, personal communication) was applied.^13^ The pain level was evaluated based on the answer to the FIQR question, scored on an 11-point numeric rating scale (from 0 to 10). The number of tender points was evaluated based on the 1990 ACR classification criteria.^11^

### Traditional Cardiovascular Risk Factors

Smoking and drinking habits were documented for all subjects. Body mass index (BMI) was calculated according to the standard equation (weight in kg divided by the square of body height in m²). Blood pressure was measured using the automatic blood pressure machine (Omron No HBP 9020, Kyoto, Japan). Mean blood pressure was calculated according to the standard equation (1/3 systolic and 2/3 diastolic pressures). Blood levels of fasting sugar (FBS), total cholesterol, low-density lipoprotein cholesterol (LDL-C), high-density lipoprotein cholesterol (HDL-C), and triglycerides were all determined with spectrophotometry (the Abbott Alinity I-series system). Hemoglobin A1c (HbA1c) was determined with high-performance liquid chromatography.

### Carotid Intima-Media Thickness

Bilateral carotid IMT and plaque were determined ultrasonically with a predetermined protocol, using the Philips iE33 Echo machine (Amsterdam, Netherlands). Participants were kept in a supine position while applying an electrocardiography patch concomitantly. The neck of the subject was exposed and extended, with the head turning 30° toward the opposite side of measurement. Then, the IMT was measured at the right and left distal common carotid arteries (CCA) in the long axis view, and an image was obtained automatically. The ultrasound transducer frequency was set at 5-13 MHz, and the penetrating depth of the ultrasound machine was set at 4 cm. After taking an image, the experienced technician chose a 1-cm-wide region between the bulb area and CCA, and the mean values of IMT were calculated automatically.

### Metabolic Syndrome

As defined by the National Cholesterol Education Program Adult Treatment Panel III,^14^ metabolic syndrome of a subject was considered present if ≧ 3 of the following 5 criteria had been met: (a) central adiposity (waist circumference [measured at the midpoint between the lower margin of the least palpable rib and the top of the iliac crest] ≥ 90 cm for males and ≥ 80 cm for females), (b) hypertension (systolic/diastolic blood pressure ≥ 130/85 mmHg or current use of antihypertensive drugs), (c) hypertriglyceridemia (fasting TG level ≥ 150 mg/dL), (d) low HDL-C (fasting HDL-C level < 40 mg/dL (men) or < 50 mg/dL (women)), and (e) hyperglycemia (FBS ≥ 100 mg/dL or current use of antihyperglycemic drugs). Disease severity of FM, such as the FIQR score, number of tender points, and level of pain, was compared between FM patients with and without metabolic syndrome.

### Metabolic Dysfunction–Associated Steatotic Liver Disease

Sonography of the liver was performed using the Canon APLIO 500 Ultrasound System (Tochigi, Japan) and interpreted by an experienced hepatologist (CC Chen). The diagnosis of steatotic liver disease (SLD) and severity of steatosis were evaluated with the Saverymuttu criteria,^15^ as (a) mild, (b) moderate, or (c) severe.^15^ The Hamaguchi severity score for hepatic steatosis was also calculated (ranged from 0 to 6)^16^ and the score is known to be well correlated with the degree of hepatic steatosis histologically.^17^ The diagnosis of MASLD was based on the American Association for the Study of Liver Diseases guideline.^18^ The presence of SLD plus at least 1 of 5 accompanying cardiometabolic risk factors was required for its diagnosis: (a) BMI ≥ 24 kg/m^2^ or waist circumference > 90 cm (male) or 80 cm (female); (b) FBS ≥ 144 mg/dL, HbA1c ≥ 5.7%, diagnosis of diabetes mellitus (DM), or treatment for DM; (c) blood pressure ≥ 130/85 mmHg or treatment for hypertension; (d) plasma triglycerides ≥ 150 mg/dL or lipid-lowering treatment; (e) plasma HDL-C ≤ 40 mg/dL (male) or ≤ 50 mg/dL (female) or lipid-lowering treatment.

### Sensitivity Analyses

Sensitivity analyses were performed on female subjects only and FM patients diagnosed with the 1990 ACR criteria only to examine if the results change.

### Statistical Analyses

Variables are presented as medians and interquartile ranges, and categorical data are presented as percentage. Comparisons between the groups of subjects were performed using the Mann–Whitney *U*-test and chi-square test. Multivariate logistic and linear regression (after logarithmic transformation of dependent variables) were performed to compare inter-group differences, after adjustment for age and sex. The correction for multiple comparisons was not performed due to the exploratory study design. All statistical analyses were performed using SPSS software version 22.0 (IBM SPSS Corp.; Armonk, NY, USA).

## Results

### Clinical Characteristics

Clinical characteristics of the study subjects are shown in Table 1. The majority of FM patients were female. The median FIQR score was 52 in FM patients. Among the FM patients, 31 (47%) and 28 (42%) received tramadol and pregabalin, respectively.

### Comparisons of Traditional Cardiovascular Risk Factors

Fibromyalgia patients had a lower body weight, a higher waist circumference and HDL-C level, and a lower proportion of drinking and low HDL-C when compared with the controls (Table 2).

### Carotid Intima-Media Thickness

As shown in Table 2, FM patients and controls had similar carotid IMT values bilaterally.

### Metabolic Syndrome

No inter-group difference was found in the prevalence of metabolic syndrome (Table 2). Fibromyalgia patients with metabolic syndrome had a higher FIQR score (69 [IQR 46, 80] vs. 48 [IQR 30, 66], *P* < .05) and a trend toward a higher level of pain (8 [IQR 6, 9] vs. 6 [IQR 4, 8], *P* = .065) when compared with those patients without metabolic syndrome. On the other hand, FM patients with and without metabolic syndrome had a similar number of tender points (15 [IQR 12, 18] vs. 14 [IQR 11, 16].

### Metabolic Dysfunction–Associated Steatotic Liver Disease

The prevalence of MASLD was not different between groups. The Hamaguchi severity scores for hepatic steatosis appeared slightly higher in FM patients diagnosed with MASLD (Table 2).

### Multivariate Analyses

As illustrated in Table 3, FM patients had a higher proportion of central adiposity than the controls, with an odds ratio of 6.1 (95% CI: 2.9, 13.1). There appeared to be a trend toward a higher proportion of MASLD in FM patients when compared with the controls (odds ratio 1.9; 95% CI: 1.0, 3.8). The proportion of traditional cardiovascular factors and metabolic syndrome, and bilateral carotid IMT values were not different between groups after adjustment for age and sex. To be noted, in subjects diagnosed with MASLD, the Hamaguchi severity score for hepatic steatosis was higher in FM patients when compared with controls.

### Correlational Analyses

In FM patients, as illustrated in Table 4, their left carotid IMT value was weakly associated with the number of tender points. The triglyceride blood levels were weakly associated with the FIQR score. The triglyceride and total cholesterol blood levels were moderately associated with the number of tender points. The total cholesterol and LDL-C blood levels were associated with the level of pain.

### Sensitivity Analyses

In female subjects, notably, FM patients had a higher proportion of MASLD than the controls, with an odds ratio of 2.8 (95% CI: 1.3, 6.1) (Supplementary Table 1). Other results were similar to the primary analysis. After exclusion of 10 FM patients diagnosed with the 1990 ACR criteria, the results did not change significantly (Supplementary Table 2).

## Discussion

This study revealed comparable cardiovascular risk factors in FM patients when compared with the control subjects. These risk factors included metabolic syndrome and carotid IMT. The FM group had more subjects with central adiposity, and hepatic steatosis was more severe in those diagnosed with MASLD. In female subjects, the FM group had more subjects with MASLD. In FM patients, their disease severity was associated with the left IMT value and the blood levels of total cholesterol, LDL-C, and triglycerides.

There is controversy in regard to the cardiovascular risk of FM patients. A recent meta-analysis of 1500 FM patients revealed a more sedentary lifestyle in these patients in comparison with the general population.^2^ Both the gait and balance are severely impaired in these patients, leading to functional disability.^19^ In addition, these patients have higher levels of inflammatory cytokines,^20^^,^^21^ which likely contribute to a higher cardiovascular risk.^22^ A few studies have addressed the issue of higher cardiovascular risks. In terms of traditional cardiovascular risk factors, Cure et al^8^ studied 174 FM patients, and they only found a smoking rate higher than those of controls. Loevinger et al^23^ studied 109 FM patients, and they found higher levels of glycosylated hemoglobin, serum triglyceride, blood pressure, and LDL-C. Triantafyllias et al^5^ studied 99 FM patients and found a higher BMI and a greater proportion of them using antihypertensive drugs. A higher proportion of dyslipidemia is also reported in FM patients by several research groups.^6^^,^^24^ On the contrary, Bölük et al^4^ and Gunturk et al^7^ found no difference in cardiovascular risk factors between FM patients and controls. Finally, based on the atherosclerotic cardiovascular disease calculator, FM patients aged 40-59 years had a higher lifetime risk when compared with the controls (odds ratio: 1.56, 95% CI: 1.01-2.42).^6^ It was found in the present study, with multivariate analyses, that there was no difference in the traditional cardiovascular risk factors between FM patients and controls.

In terms of the burden of atherosclerosis, Bölük et al^4^ found greater values in both carotid IMT among FM patients when compared with controls, but the authors did not survey their smoking habits and hypertension. Triantafyllias et al^5^ found that their FM patients had increased aortic stiffness, represented by carotid femoral pulse wave velocity (cfPWV). In another study on FM patients, a higher cfPWV and aortic stiffness index, and a lower aortic distensibility value were observed, although the lipid profile was not studied in the controls.^7^ Contrary to the above findings, a greater burden of atherosclerosis in the FM patients was not observed based on carotid IMT values.

Metabolic syndrome refers to the clustering of metabolic risk factors for type 2 diabetes and cardiovascular disease. These factors include abdominal obesity, hyperglycemia, dyslipidemia, and hypertension.^25^ Central to its contribution to cardiovascular risk is the underlying insulin resistance.^26^ Metabolic syndrome is a well-recognized risk factor for developing cardiovascular diseases. Researchers reported a 4 to 5-fold higher prevalence rate of metabolic syndrome in FM patients, and its presence was correlated with greater disease severity.^23^^,^^27^ In addition, a study on 70 FM patients reported a higher level of insulin resistance when compared with controls.^28^ Despite these reports, more central adiposity in the FM patients was found when compared with controls. This finding is compatible with a recent meta-analysis supporting a potential relationship between obesity and FM symptoms.^29^ Interestingly, greater disease severity was found, represented by the FIQR score, in FM patients with metabolic syndrome when compared with those patients without.

Metabolic dysfunction–associated steatotic liver disease is frequently associated with metabolic syndrome and potentially progresses to liver cirrhosis.^30^ Notably, FM symptoms are reportedly associated with metabolic dysfunction–associated steatohepatitis in patients who developed liver cirrhosis.^31^ A recent study, on the other hand, found a lower prevalence of MASLD in FM patients when compared with controls, likely related to their lower prevalence of obesity.^8^ In the FM patients, the severity of hepatic steatosis appeared higher than that of the controls. In addition, the proportion of MASLD in female FM patients was higher than in the female controls.

Disease severity of FM was not found to be associated with carotid IMT values or aortic stiffness in a previous study.^4^^,^^5^ However, the FIQR score was an important contributor in the predictive model, based on machine learning, for cardiovascular disease in FM patients.^6^ Gunturk et al^7^ reported very strong positive correlations between the cfPWV value and number of tender points, Visual Analogue Scale pain score, and FIQR (all correlational coefficients > 0.9). In accordance with the previous findings, an association between left carotid IMT value and the number of tender points in FM patients was observed. Interestingly, in the FM patients, blood levels of total cholesterol, LDL-C, and triglycerides were associated with disease severity measures (FIQR, number of tender points, and level of pain), whereas a previous study on FM patients did not find an association between serum lipid profile (total cholesterol and LDL-C) and disease severity.^24^

Our study has some limitations. First, the FM patients were under medication, which might have affected the results with respect to disease severity. Second, the cross-sectional study was not able to longitudinally evaluate the association between cardiovascular risk and FM. Third, missing data in the study may introduce bias. Fourth, the sonographic grading of MASLD could well predict hepatic steatosis, but could not reflect hepatic fibrosis.^15^ Fifth, a priori power analysis was not conducted in the observational study. Sixth, most of the FM patients were diagnosed with the 1990 ACR classification criteria rather than the 2016 revised ACR criteria, since this study was conducted since January 2016. The 1990 ACR criteria have been recognized as stricter than the 2016 criteria, such that only more severe patients are identified.^32^ In addition, these results did not change after exclusion of 10 FM patients diagnosed with the 2016 ACR criteria. Lastly, the controls were recruited from individuals undergoing health examinations. They were not necessarily healthy and could have had significant diseases. Nevertheless, it was believed that they could represent the general population, and the multivariate analyses had adjusted for their baseline characteristics.

In conclusion, no difference was found in the proportion of metabolic syndrome between FM patients and controls, despite a higher proportion of central adiposity in FM patients. Fibromyalgia patients might have more severe hepatic steatosis if MASLD was present. Fibromyalgia patients with metabolic syndrome might have more severe symptoms when compared with those patients without. Disease severity of FM might be associated with an increased left IMT value and lipid blood levels.

## Figures and Tables

**Table 1.** Baseline Characteristics

Characteristics	FM Patients (n = 66)	Controls (n = 116)	*P*
Age, median (IQR) (years)	48 (40, 59)	46 (39, 57)	.56
Female sex, n (%)	59 (89)	57 (49)	<.001
Number of tender points, median (IQR)^a^	14 (11, 17)	NA	
FIQR, median (IQR)	52 (30, 69)	NA	
Level of pain, median (IQR)	7 (4, 8)	NA	
Medications, n (%)			
Pregabalin	28 (42)	NA	
Duloxetine	2 (3)	NA	
Tramadol	31 (47)	NA	
Amitriptyline	4 (6)	NA	

FM, fibromyalgia; FIQR, Revised Fibromyalgia Impact Questionnaire; IQR, interquartile range; NA, not available.

^a^Two FM patients did not have number of tender points recorded.

**Table 2.** Cardiovascular Profile of Study Subjects

Cardiovascular Profile	FM Patients (n = 66)	Controls (n = 116)	*P*
Body weight, median (IQR) (kg)	58 (50, 66)	63 (56, 74)	.003
BMI, median (IQR)	23 (20, 26)	24 (21, 26)	.32
Smoking, n (%)	7 (11)	16 (14)	.53
Drinking, n (%)	3 (5)	17 (15)	.036
Waist circumference, median (IQR) (cm)	84 (75, 94)	79 (72, 87)	.017
MBP, median (IQR) (mmHg)	88 (77, 97)	88 (81, 99)	.30
Hypertension, n (%)	24 (36)	43 (37)	.92
FBS, median (IQR) (mg/dL)^a^	89 (84, 98)	89 (84, 96)	.70
HbA1c, median (IQR) (%)^b^	5.6 (5.3, 5.8)	5.5 (5.3, 5.7)	.73
Hyperglycemia, n (%)	12 (36)	21 (18)	.99
HDL-C, median (IQR) (mg/dL)	62 (53, 74)	56 (47, 70)	.023
Low HDL-C, n (%)	9 (14)	37 (32)	.006
LDL-C, median (IQR) (mg/dL)^c^	118 (98, 143)	116 (99, 137)	.52
High LDL-C, n (%)^b^	26 (41)	50 (43)	.75
Total cholesterol, median (IQR) (mg/dL)	199 (171, 231)	197 (172, 219)	.48
High total cholesterol, n (%)	31 (47)	49 (42)	.54
Triglyceride, median (IQR)	97 (64, 145)	95 (67, 137)	.99
Hypertriglyceridemia, n (%)	14 (21)	25 (22)	.96
Left carotid IMT median (IQR) (mm)^d^	0.53 (0.49, 0.58)	0.58 (0.50, 0.68)	.078
Left carotid plaque, n (%)^d^	11 (18)	8 (22)	.62
Right carotid IMT, median (IQR) (mm)^e^	0.53 (0.48, 0.64)	0.60 (0.50, 0.71)	.052
Right carotid plaque, n (%)^e^	6 (10)	10 (28)	.021
Metabolic syndrome, n (%)	14 (21)	25 (22)	.96
SLD, n (%)^f^	41 (71)	74 (64)	.36
MASLD, n (%)^f^	35 (53)	55 (47)	.47
Saverymuttu score, median (IQR)^f^	1 (1, 2)	1 (1, 2)	.18
Hamaguchi score, median (IQR)^f^	2 (1, 3)	2 (1, 2)	.012

BMI, body mass index; FBS, fasting blood sugar; FM, fibromyalgia; HbA1c, hemoglobin A1c; HDL-C, high-density lipoprotein cholesterol; IMT, intima-media thickness; IQR, interquartile range; LDL-C, low-density lipoprotein cholesterol; MASLD, metabolic dysfunction–associated steatotic liver disease; MBP, mean blood pressure; SLD, steatotic liver disease.

^a^FBS was not determined in 1 FM patient.

^b^HbA1c was not determined in 2 FM patients and 20 controls.

^c^LDL-C was not determined in 2 FM patients.

^d^Left carotid sonography was not measured in 5 FM patients and 80 controls.

^e^Right carotid sonography was not measured in 5 FM patients and 80 controls.

^f^Liver sonography was not performed in 8 FM patients. Severity scores were calculated in those subjects with MASLD diagnosed.

**Table 3.** Multivariate Analyses on the Cardiovascular Risk Factors in Fibromyalgia Patients When Compared with the Controls

Multivariate Analyses	Fibromyalgia Patients
Logistic regression, odds ratio (95% CI)	
Smoking	1.5 (0.5, 4.8)
Drinking	0.7 (0.2, 3.1)
Central adiposity	6.1 (2.9, 13.1)^***^
Hypertension	1.6 (0.7, 3.4)
Hyperglycemia^a^	0.7 (0.3, 1.8)
Low HDL-C	0.7 (0.3, 1.9)
High LDL-C^b^	1.2 (0.6, 2.5)
Hypercholesterolemia	1.1 (0.5, 2.1)
Hypertriglyceridemia	2.1 (0.8, 5.3)
Left carotid plaque^c^	1.9 (0.5, 7.8)
Right carotid plaque^d^	1.3 (0.3, 6.5)
Metabolic syndrome	1.8 (0.7, 4.6)
MASLD^e^	1.9 (1.0, 3.8)
Linear regression, regression coefficient (95% CI)	
Log (left carotid IMT)^c^	−0.008 (−0.087, 0.070)
Log (right carotid IMT)^d^	−0.006 (−0.092, 0.080)
Log (Saverymuttu score)^e^	−0.111 (−0.284, 0.062)
Log (Hamaguchi score)^e^	0.338 (0.082, 0.595)^*^

FM, fibromyalgia; HDL-C, high-density lipoprotein cholesterol; IMT, intima-media thickness; IQR, interquartile range; LDL-C, low-density lipoprotein cholesterol; MASLD, metabolic dysfunction–associated steatotic liver disease.

^a^FBS was not determined in 1 FM patient.

^b^LDL-C was not determined in 2 FM patients.

^c^Left carotid sonography was not measured in 5 FM patients and 80 controls.

^d^Right carotid sonography was not measured in 5 FM patients and 80 controls.

^e^Liver sonography was not performed in 8 FM patients. Severity scores were calculated in those subjects with MASLD diagnosed.

*P < .05.

***P < .005.

**Table 4.** Correlations Between Cardiovascular Risk Factors and Disease Severity Measures in Fibromyalgia Patients

Correlation Coefficients	FIQR Score	Number of Tender Points^a^	Levels of Pain
BMI	0.11	−0.02	0.03
Waist conference	0.09	0.01	0.04
MBP	0.01	−0.13	−0.04
Fasting blood sugar^b^	−0.12	0.16	−0.02
HbA1c^c^	−0.15	0.09	−0.05
HDL-C	−0.21	−0.12	−0.14
LDL-C^d^	0.22	0.24	0.26^*^
Total cholesterol	0.21	0.30^*^	0.30^*^
Triglyceride	0.25^*^	0.41^***^	0.20
Left carotid IMT^e^	−0.10	0.27^*^	−0.14
Right carotid IMT^f^	0.20	0.21	0.20
Saverymuttu score^g^	0.26	0.22	0.24
Hamaguchi score^g^	0.12	0.22	0.19

BMI, body mass index; FIQR, the Revised Fibromyalgia Impact Questionnaire; HbA1c, hemoglobin A1c; HDL-C, high-density lipoprotein cholesterol; IMT, intimal media thickness; LDL-C, low-density lipoprotein cholesterol; MBP, mean blood pressure; MASLD, metabolic dysfunction–associated steatotic liver disease.

^a^Number of tender points was not determined in 2 fibromyalgia patients.

^b^Fasting blood sugar was not determined in 1 fibromyalgia patient.

^c^HbA1c was not determined in 2 fibromyalgia patients.

^d^LDL-C was not determined in 2 fibromyalgia patients.

^e^Left carotid sonography was not measured in 5 fibromyalgia patients.

^f^Right carotid sonography was not measured in 5 fibromyalgia patients.

^g^Liver sonography was not performed in 8 fibromyalgia patients. Severity scores were calculated in those subjects with MASLD diagnosed.

*P < .05.

***P < .005.

**Supplementary Table 1.** Multivariate analyses on the cardiovascular risk factors in female FM patients when compared with the female controls

Multivariate analyses	Fibromyalgia patients
Logistic regression, odds ratio (95% confidence interval)	
Smoking	2.2 (0.4, 12.4)
Drinking	3.6 (0.3, 37.1)
Central adiposity	8.1 (3.3, 19.6)^***^
Hypertension	1.2 (0.5, 3.0)
Hyperglycemia	0.9 (0.3, 2.1)
Low HDL-C	0.7 (0.5, 5.6)
High LDL-C^a^	1.4 (0.6, 3.3)
Hypercholesterolemia	1.1 (0.5, 2.1)
Hypertriglyceridemia	1.3 (0.6, 2.7)
Left carotid plaque^b^	1.1 (0.2, 4.8)
Right carotid plaque^c^	0.6 (0.1, 4.2)
Metabolic syndrome	2.7 (0.9, 8.4)
MASLD^e^	2.8 (1.3, 6.1)^**^
Linear regression, regression coefficient (95% confidence interval)	
Log (left carotid IMT)^b^	-0.011 (-0.093, 0.072)
Log (right carotid IMT)^c^	0.049 (-0.033, 0.131)
Log (Saverymuttu score)^d^	-0.020 (-0.251, 0.211)
Log (Hamaguchi score)^d^	0.366 (0.073, 0.659)^*^

^a^LDL-C was not determined in 2 FM patients.

^b^Left carotid sonography was not measured in 5 FM patients and 42 controls.

^c^Right carotid sonography was not measured in 5 FM patients and 42 controls.

^d^Liver sonography was not performed in 7 FM patients. Severity scores were calculated in those subjects with MASLD diagnosed.

FM, fibromyalgia; HDL-C, high-density lipoprotein cholesterol; IMT, intima-media thickness; IQR, interquartile range; LDL-C, low-density lipoprotein cholesterol; MASLD, metabolic dysfunction-associated steatotic liver disease.

^*^P<.05; ^**^P<.01; ^***^P<.005.

**Supplementary Table 2.** Multivariate analyses on the cardiovascular risk factors in FM patients diagnosed with the 1990 ACR criteria when compared with the controls

Multivariate analyses	Fibromyalgia patients
Logistic regression, odds ratio (95% confidence interval)	
Smoking	2.1 (0.6, 7.0)
Drinking	0.3 (0.0, 2.8)
Central adiposity	5.2 (2.4, 11.3)^***^
Hypertension	1.3 (0.6, 2.8)
Hyperglycemia	0.8 (0.3, 2.0)
Low HDL-C	0.9 (0.4, 2.3)
High LDL-C^a^	1.4 (0.7, 3.0)
Hypercholesterolemia	1.0 (0.5, 2.1)
Hypertriglyceridemia	2.4 (0.9, 6.3)
Left carotid plaque^b^	1.2 (0.3, 5.2)
Right carotid plaque^c^	0.7 (0.1, 3.8)
Metabolic syndrome	2.0 (0.8, 5.1)
MASLD^e^	1.6 (0.8, 3.3)
Linear regression, regression coefficient (95% confidence interval)	
Log (left carotid IMT)^b^	-0.011 (-0.093, 0.070)
Log (right carotid IMT)^c^	-0.009 (-0.100, 0.083)
Log (Saverymuttu score)^d^	-0.112 (-0.301, 0.077)
Log (Hamaguchi score)^d^	0.341 (0.074, 0.609)^*^

^a^LDL-C was not determined in 2 FM patients.

^b^Left carotid sonography was not measured in 5 FM patients and 80 controls.

^c^Right carotid sonography was not measured in 5 FM patients and 80 controls.

^d^Liver sonography was not performed in 8 FM patients. Severity scores were calculated in those subjects with MASLD diagnosed.

FM, fibromyalgia; HDL-C, high-density lipoprotein cholesterol; IMT, intima-media thickness; IQR, interquartile range; LDL-C, low-density lipoprotein cholesterol; MASLD, metabolic dysfunction–associated steatotic liver disease.

^*^P<.05; ^ ***^P<.005.

## Data Availability

The data that support the findings of this study are available on request from the corresponding author.
